# Temperature-induced reactivation of Marek's disease virus-transformed T cells *ex vivo*

**DOI:** 10.3389/fvets.2023.1145757

**Published:** 2023-03-08

**Authors:** Yung-Tien Tien, Haji Akbar, Keith William Jarosinski

**Affiliations:** Department of Pathobiology, College of Veterinary Medicine, University of Illinois at Urbana-Champaign, Urbana, IL, United States

**Keywords:** Marek's disease (MD), reactivation, herpesvirus, chicken, transformation

## Abstract

Marek's disease virus (MDV) establishes latency in chicken T lymphocytes that can lead to T cell transformation and cancer. Transformed Marek's disease chicken cell lines (MDCCs) can be expanded *ex vivo* and provide a valuable model to study latency, transformation, and reactivation. Here, we developed MDCCs from chickens infected with MDV that fluoresce during lytic replication and reactivation. Sodium butyrate treatment increased fluorescent protein expression as evidenced by fluorescent microscopy, flow cytometry, and western blotting; however, it caused significant apoptosis and necrosis. Treatment of MDCCs by decreasing the temperature resulted in robust MDV reactivation without significant induction of apoptosis and necrosis. Furthermore, MDV reactivation was significantly affected by the time in culture that can affect downstream reactivation analyses. In all, our data show that fluorescent protein expression during reactivation is a robust tool to examine viral replication in live cells *ex vivo*, and temperature treatment is an efficient technique to induce reactivation without punitive effects on cell viability seen with chemical treatment.

## 1. Introduction

Marek's disease is a lymphoproliferative disease in chickens. It is caused by Marek's disease virus (MDV) or *Gallid alphaherpesvirus* 2. The high mortality and morbidity of Marek's disease cause substantial economic losses in poultry meat and egg production worldwide, estimated at 1 billion USD every year (1). Despite numerous commercial vaccines available, MDV has evolved over time to “breakthrough” vaccine protection (2). Today, sporadic outbreaks still happen globally (3, 4). The most common cause of death in chickens with Marek's disease is from lymphomas similar to Burkitt's lymphoma caused by Epstein-Barr herpesvirus in humans. MDV is transmitted through inhalation of contaminated dust and dander (5, 6), where pulmonary macrophages and B cells are first infected (7) and transport the virus to lymphoid tissues such as the bursa of Fabricius, spleen, and thymus where T cells are infected (8). Ultimately, the virus establishes latency in T cells, where some can be transformed into neoplastic lymphoma cells promoted by the MDV oncoprotein, Meq (9). MDV-transformed chicken cells (MDCC) have been isolated and cultured historically since 1973 when Akiyama et al. (10) described MOB1.

Latency is an important hallmark of herpesviruses. MDCCs contain MDV that is latent in the cell, with the viral genome maintained by integration into the host telomeres (11, 12). Few viral genes are expressed, apart from latency-associated transcripts (LATs). For example, LATs like miR-M7-5p can degrade immediate early gene expressions, such as *ICP4* and *ICP27*, to suppress viral gene expression (13, 14). Reactivation usually happens in response to cell damage or stress, such as apoptosis, hypoxia, or metabolic stress, to initiate lytic replication (15). A recent study showed that acute exposure to 1% O_2_ can cause upregulation of late MDV genes *UL47* (VP13/14), *UL49* (VP22), and *UL27* glycoprotein B (gB), indicating reactivation (16). Chemicals such as sodium butyrate have been utilized to induce reactivation in herpesviruses (17), including MDV (18–20). Other than chemical treatment, serum starvation and decreasing temperature can induce MDV reactivation (21–24).

Many laboratories have developed different models to study MDV reactivation in MDCCs; however, the lack of tools to distinguish reactivating viruses in live cells has hindered this progress. In 2012, a recombinant MDV (rMDV) in which the late viral protein, pUL47, was tagged with an enhanced green fluorescent protein (eGFP) termed vUL47eGFP (25) provided this tool. Unlike other attempts at “tagging” viral proteins that typically resulted in attenuated MDV (26–32), vUL47eGFP was not affected by the fluorescent tag and has been used by our group and others to identify infected cells *in vivo and ex vivo*. Two groups have utilized vUL47eGFP to generate MDCCs (33, 34) with success in identifying MDV at late stages of replication. However, since pUL47 is a late viral protein, only 1–2% of viable cells spontaneously expressed low levels of pUL47eGFP. Using another rMDV in which the early viral protein, repeat long open reading frame (ORF) 4 (RLORF4), was tagged with monomeric red fluorescent protein (mRFP), we found this tag also did not result in attenuation (35), similar to vUL47eGFP. Importantly, this virus (vRLORF4mRFP) allowed us to identify cells during the early stage of replication based on RLORF4mRFP expression (35).

During our studies evaluating reactivation in MDCCs derived from vRLORF4mRFP-infected chickens, we found sodium butyrate treatment resulted in both reactivation and cell death; therefore, we sought to find a less toxic method to induce reactivation. Here, we developed an *ex vivo* reactivation model using a previously described fluorescent virus to identify reactivating cells using decreased temperature treatment. The core body temperature of chickens is approximately 41°C, while the surface body temperature can fluctuate in ambient temperatures and range from 20 to 40°C (36). Importantly, fully productive lytic replication of MDV occurs in the skin of infected chickens, suggesting temperature may play a role in virus replication. We hypothesized treatment of cells at lower than traditional temperatures may induce lytic replication in MDCCs. Our results showed that simple temperature treatment induced significant reactivation without substantial cell death compared to sodium butyrate.

## 2. Materials and methods

### 2.1. Cell culture and cells

#### 2.1.1. Cells

Chick embryo cells (CECs) were prepared from 10 to 11-day-old specific-pathogen-free chicken embryos obtained from the University of Illinois at Urbana-Champaign Poultry Farm following standard methods (37). CECs were seeded in a growth medium consisting of M20 media [Medium 199 (Cellgro, Corning, NY, USA) supplemented with 10% tryptose-phosphate broth (TPB), 0.63% NaHCO3 solution, antibiotics (100 U/ml penicillin and 100 μg/ml streptomycin)], and 4% fetal bovine serum (FBS). Confluent CECs were maintained in M20 media supplemented with 10% tryptose-phosphate broth (TPB), 0.63% NaHCO3 solution, and antibiotics (100 U/ml penicillin and 100 μg/ml streptomycin) and 0.2 % FBS and maintained at 38°C in a humidified atmosphere of 5% CO_2_.

DT40 chicken B lymphoid cells were purchased from ATCC (CRL-2111) and maintained in a 1:1 mixture of Leibovitz L-15 and McCoy 5A (LM) media (Gibco, Gaithersburg, MD, USA) supplemented with 10% FBS (LM10) and antibiotics. To generate a positive monomeric red fluorescent protein (mRFP) control for our studies, 5 × 10^6^ DT40 cells were transfected with 5 μg pDsRed1-N1 (Clontech Laboratories, Inc., San Jose, CA, USA) by electroporation using a Nucleofector I device (Lonza, Basel, CH), following the provided protocols for the Amaxa Human B Cell Nucleofector Kit (VPA-1001, Lonza). The transfected DT40 cells were selected in 500 μg/ml G418 disulfate (Invitrogen, Carlsbad, CA, USA) immediately after electroporation and selected for seven days, then maintained at 200 μg/ml in LM10 media with antibiotics.

#### 2.1.2. MDV-transformed chicken cells (MDCCs)

The recombinant (r)MDV used in this report has been previously described (35, 38) and MDCCs were generated from previously published animal experiments (38). For both rMDV, mRFP was fused to the MDV-specific early gene, RLORF4, termed vRLORF4mRFP (35) in the RB-1B strain bacterial artificial chromosome clone. The other rMDV additionally had the immediate early ICP27 (*UL54*) N-terminally tagged with a 3 × Flag epitope and designated vRLORF4mRFP/3 × Flag54 (38).

To generate MDCCs, tumors were collected from MDV-infected chickens and smashed through a 70 μm EASYstrainer (Greiner Bio-One, Monroe, NC, USA) with phosphate-buffered saline (PBS) buffer. The cell pellets were collected after centrifugation with 400 × *g* for 5 min and single-cell suspensions were prepared using Histopaque 1077 (Sigma-Aldrich, St. Lois, MO, USA) centrifugation at 400 × *g* for 15 min. Purified mononuclear cells were cultured in LM media supplemented with 10% of FBS and 8% of chicken serum and antibiotics at 41°C with 5% CO_2_. After 2 weeks, the chicken serum concentration was gradually reduced to FBS only. A total of four RLORF4mRFP-tagged MDCCs were established and used in this study, one expressing RLORF4mRFP, and three expressing RLORF4mRFP and 3 × Flag-tagged UL54. All MDCCs were generated from specific pathogen-free MD-susceptible Pure Colombian chickens (Table 1). The major histocompatibility complex haplotype of Pure Columbian chickens has not been defined but is suggested to be B6-like (39).

**Table 1:** Marek's disease virus (MDV)-transformed chicken cells (MDCCs) used in this study.

**Name^a^**	**Virus^b^**	**Tissue and time^c^**	**Time in culture^d^**	**CD4 (%)**	**CD8 (%)**	**Bu1 (%)**
KJ1072	vRLORF4mRFP	Spleen (28 dpi)	27 wk	99.25 ± 0.05	0.09 ± 0.03	0.1 ± 0.00
KJ1063	vRLORF4mRFP/3 × Flag54	Kidney (34 dpi)	27 wk	99.41 ± 0.13	0.08 ± 0.05	0.24 ± 0.02
WV5113	vRLORF4mRFP/3 × Flag54	Kidney (28 dpi)	30 wk	99.98 ± 0.02	0.08 ± 0.04	0.08 ± 0.08
WV6847	vRLORF4mRFP/3 × Flag54	Kidney (25 dpi)	15 wk	95.49 ± 3.84	0.10 ± 0.05	0.07 ± 0.04

^a^MDCC designation.

^b^Infecting virus.

^c^Tissue from which tumor was collected and time of collection after experimental infection in days post-infection (dpi).

^d^Weeks (wk) in culture during characterization.

### 2.2. Immunofluorescence assays

IFAs were performed as previously described (40). Briefly, MDCCs were fixed with PFA buffer (2% paraformaldehyde, 0.1% Triton X-100) for 15 min and then washed twice with PBS. Cells were blocked in 10% neonatal calf serum and stained with mouse monoclonal antibody (mAb) H19 (41) or IAN86.17 (42) to detect phosphoprotein 38 (pp38) and gB, respectively, plus goat anti-mouse IgG-Alexa Fluor 488 secondary antibody (Molecular Probes, Eugene, OR). Cells were then incubated with 3 μM DAPI (4',6-Diamidino-2-Phenylindole, Dihydrochloride; Sigma-Aldrich) for 5 min to stain DNA, then washed twice. Stained cells (~10,000 cells) were mounted on a slide and a glass-coverslip was gently laid onto the cells. Fluorescent protein expression was directly visualized using a red filter and images were obtained using an EVOS FL Cell Imaging System (Thermo Fisher Scientific, Waltham, MA, USA) and compiled using Adobe Photoshop version 21.0.1.

### 2.3. Reactivation assays

A plaque formation assay was performed to measure the number of cells reactivating infectious MDV (22). Briefly, 10^3^ to 10^5^ MDCCs were seeded on primary CECs, and the number of plaque-forming units was enumerated 5–7 days later. The infected cells were washed once with PBS, fixed/permeabilized with PFA buffer for 15 min, and washed twice with PBS. Anti-MDV chicken sera and goat anti-chicken IgY-Alexa Fluor 488 or 568 secondary antibody (Molecular Probes) were used. All plaques were counted manually using an EVOS FL Cell Imaging System and the average plaque forming units were enumerated.

For testing different reactivation parameters, MDCCs were separated from cellular debris using Histopaque 1077 purification. Live cells were resuspended at 2 x 10^6^ cells/ml LM10 and kept at 41°C overnight. The next day, cells were treated with temperature change or sodium butyrate. For temperature treatment, the cells were incubated at 32°C and for chemical treatment, cells were incubated at the standard temperature (41°C) with 3 mM of sodium butyrate.

### 2.4. Flow cytometry

A Cytek Aurora flow cytometer was used for the analysis. Eight million cells were harvested after reactivation treatments as single-cell suspensions by passing the cells through 70 μm EASYstrainers at the concentration of 1 x 10^6^ cells/ml. For characterization of MDCCs, cells were stained with anti-chicken CD4-, CD8-, and Bu1-Alexa Fluor 647 antibodies (Southern Biotech, AL, USA) at 1:1000 dilutions. For all samples, DAPI staining solution (3 μM DAPI, 100 mM Tris pH 7.4, 150 mM NaCl, 1 mM CaCl_2_, 0.5 mM MgCl_2_, 0.1% Nonidet P-40) was added to the single-cell suspensions for 15 min before analyzing by flow cytometry to measure cell viability. Flow cytometry examining mRFP are shown after gating on lymphocytes, followed by DAPI negative for live cells. Ten thousand gated cells were acquired per sample and the data was analyzed using FlowJo software version 10 (Ashland, OR, USA).

### 2.5. Reverse transcription (RT)-quantitative polymerase chain (qPCR) assays

To measure viral gene expression in MDCCs, RT-qPCR assays were used as previously described (38). Briefly, total RNA was collected from 4 x 10^6^ cells for each cell line after 8 and 24 h of treatment using RNA STAT-60 (Tel-Test, Inc., Friendswood, TX, USA) using the manufacturer's instructions. RNA was DNase-treated using a Turbo DNA-free kit from Thermo Fisher Scientific using the manufacturer's instructions. RT was performed with 10 μg of DNase-treated total RNA using the High Capacity cDNA Reverse Transcription Kit (Thermo Fisher Scientific). One-hundred microliter reactions were carried out according to the manufacturer's instructions with random hexamer primers. The reaction mixture was incubated at 25°C for 10 min, then 37°C for 120 min, followed by 85°C for 5 min.

To amplify cDNA in RT-qPCR assays, 2 × Power SYBR Green PCR Master Mix (Thermo Fisher Scientific) was used. Quantification of MDV-specific transcripts was performed using previously described specific primers for each respective MDV transcript and chicken glyceraldehyde 3-phosphate dehydrogenase (GAPDH) as a normalizing control (38, 43). Briefly, 3 μl of the cDNA mixture was used in 20 μl volumes containing 50 μM of forward and reverse primers and standard thermal cycling conditions were used (38). All RT-qPCR assays were performed using an Applied Biosystems QuantStudio 3 Real-Time PCR System (Thermo Fisher Scientific). The results were analyzed using QuantStudio Design & Analysis Software v1.4.2 supplied by the manufacturer.

### 2.6. Viral DNA replication kinetics in cell culture

To determine the viral DNA replication kinetics of MDV in MDCCs, qPCR assays were used. MDCCs were prepared in 6-well tissue culture plates at a concentration of 10 × 10^6^/ml and collected by centrifugation at 200 × *g* for 5 min. Total DNA was collected from the cell pellets at 24, 36, and 48 h following reactivation, using DNA STAT-60 reagent (Tel-Test, Inc.). Quantification of MDV genomic copies in MDCCs was performed using primers and probes to MDV ICP4 and chicken inducible nitric oxide synthatase in duplex reactions as previously described (40). All qPCR assays were performed as absolute quantification using standard curves in an Applied Biosystems QuantStudio 3 Real-Time PCR System (Thermo Fisher Scientific), and the results were analyzed using the QuantStudio Design & Analysis Software v1.4.2. The coefficient of regression was >0.99 for all standard curves generated.

### 2.7. Western blotting

Western blot analyses were performed essentially as previously described (44). To detect the relative level of MDV infection, mouse mAb H19 (45) was used at 1:10,000 dilution to detect MDV pp38. To detect RLORF4 tagged with mRFP, anti-mRFP polyclonal antibody (ab62341; Abcam, Cambridge, MA, USA) was used at 1:2000 dilution. Anti-Flag M2 mAb (F1804, Sigma-Aldrich) were used at the manufacturer's recommended dilutions to detect 3 × Flag-tagged pICP27 (UL54). For protein loading controls, anti-GAPDH (GA1R; Thermo Scientific) and anti-β-Actin (ACTNO5; Abcam) mAb were used at their recommended dilutions. Secondary anti-mouse or rabbit IgG-peroxidase conjugate was purchased from GE Healthcare (Piscataway, NJ, USA). The SuperSignal West Pico Chemiluminescent Substrate kit from Thermo Fischer Scientific (Rockford, IL, USA) was used to detect antigens utilizing the manufacturer's instructions. Images were obtained using a FluorChem R imaging system (ProteinSimple, CA, USA) in 8-bit format. Protein bands were quantified using ImageJ software (version 1.6) for densitometric analysis by comparing the relative ratios of viral protein to GAPDH using the technique described on the ImageJ website (https://imagej.nih.gov/ij/docs/).

### 2.8. Necrosis and apoptosis assay

Four million cells of treated MDCCs were collected after 24 h of reactivation treatment by centrifugation at 200 x *g* for 5 min, and the cell pellets were washed twice in 100 μl of annexin-binding buffer (10 mM HEPES, 140 mM NaCl, 2.5 mM CaCl2, pH 7.4). To detect apoptotic cells, Annexin V conjugated with FITC (BD Pharmingen, San Diego, CA, USA) was used at the manufacturer's recommended dilutions for 15 min at room temperature. To differentiate necrotic cells, cells were also treated with DAPI staining solution for 15 min. The MDCCs were washed twice with PBS and kept on ice before analysis using a Cytek Aurora flow cytometer. MDCCs were gated on lymphocytes, followed by an mRFP gate, and then FITC and DAPI gates. A total of 10 × 10^3^ gated cells were acquired per sample and the data was subjected to analysis using FlowJo software.

### 2.9. Statistical analyses

IBM SPSS Statistics Version 27 software (SPSS Inc., Chicago, IL, USA) was used for statistical analyses. The normalized data for qPCR (viral replication) and RT-qPCR (viral gene transcription) were analyzed using two-way ANOVA and one-way ANOVA, respectively, followed by Tukey's *post hoc* tests. Flow cytometry data was analyzed using FlowJo version 10 and tested for distribution using both Kolmogorov–Smirnov and the Shapiro–Wilk tests. Each sample was tested at three different time points; therefore, Friedman's Test was used for two-factor analysis followed by Nemenyi *post hoc* tests using R (Studio); statistical significance was declared at *p* < 0.05. For confirmation of significant differences, the comparison of all three groups at the same time point, and individual treatments at different time points was also tested using Kruskal–Wallis H tests followed by Dunn's *post hoc* test with Bonferroni adjustment.

## 3. Results

### 3.1. Generation and characterization of MDCCs

**Figure 1:**
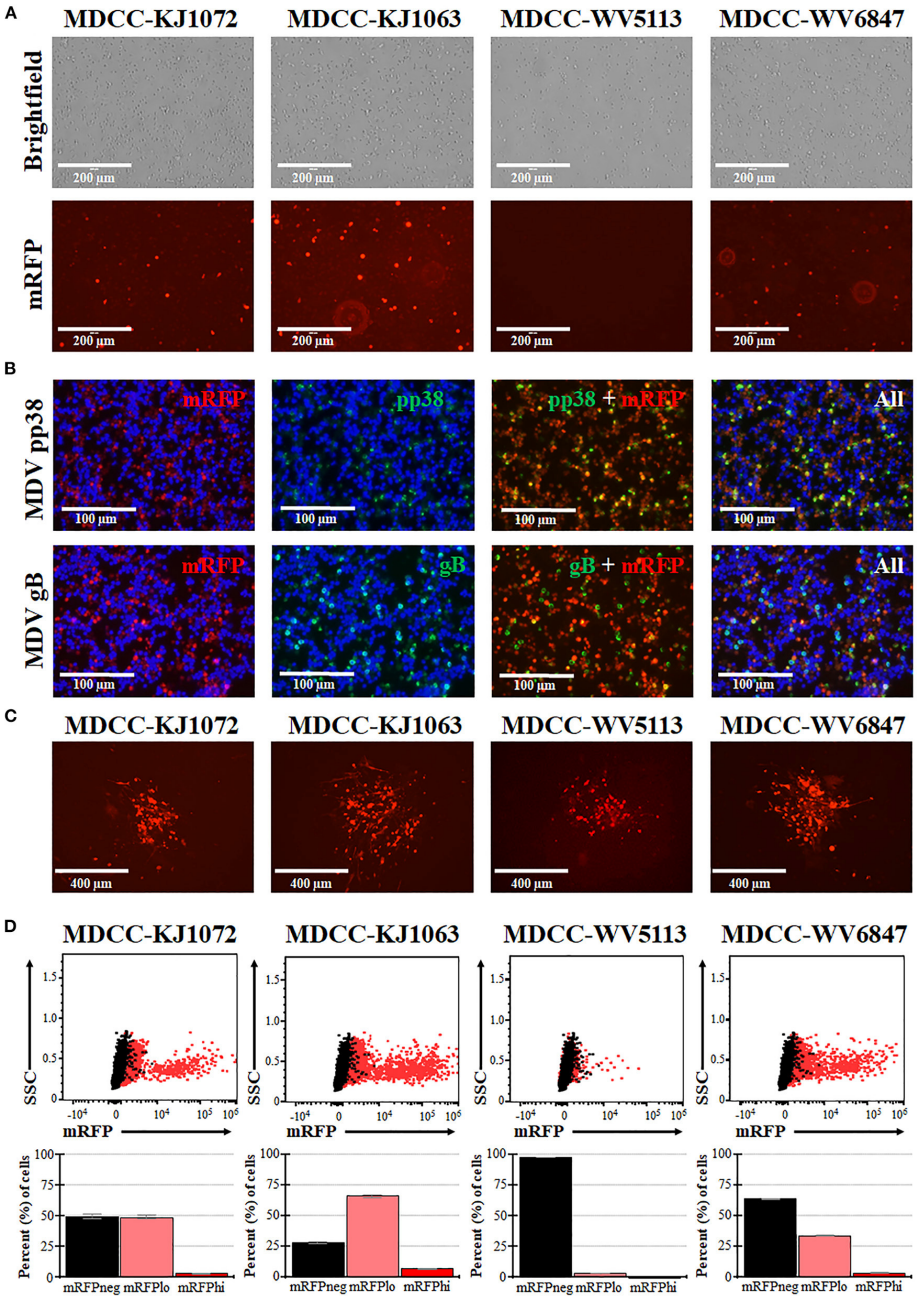
Spontaneous reactivation of MDV in MDCCs. **(A)** Light (Brightfield) and fluorescence (mRFP) microscopy images of four MDCC lines (no stain, 20×). **(B)** MDCC-KJ1063 was stained with anti-MDV pp38 or -MDV gB antibodies in IFAs. DNA (DAPI) stain is included to identify cell nuclei. **(C)** Live microscopy images of four MDCC lines expressing mRFP in CECs following reactivation assays (no stain, 10×). **(D)** Dot plots of flow cytometry data for mRFP expression in MDCCs showing mRFP negative (mRFPneg), low (mRFPlo), and high (mRFPhi) expressing cells gated on non-fluorescent DT40 cells (black dots). The mean percent cells for each population are shown below as histograms with standard deviations.

MDCCs were generated from tumors of chickens infected with rMDVs vRLORF4mRFP or vRLORF4mRFP/3 × Flag54 during a previously described study at 29- or 34-days post-infection (38). After 27 weeks *ex vivo*, > 99% of cells were CD4+ (Table 1). During the cultivation of the MDCCs *ex vivo*, we visually observed mRFP expression in live cells, particularly after Histopaque purification during weekly removal of dead cells (Figure 1A). To confirm that cells expressing mRFP were MDV positive, IFAs of MDCC-KJ1063 were used to detect the early and late viral proteins, pp38 (46) and gB (47), respectively (Figure 1B). It appeared most of the cells expressed mRFP at various levels, and a subset of those cells was also positive for pp38 or gB, confirming mRFP positive cells were infected and reactivating virus. To confirm transformed cells could reactivate virus, reactivation assays were performed, and representative plaques are shown for each cell line (Figure 1C). Expression of mRFP was maintained during virus reactivation and replication in CECs.

### 3.2. Spontaneous reactivation

After numerous fluorescent microscopy analyses, MDCCs appeared to have varying mRFP expression levels. To confirm and quantify this observation, flow cytometry was used. Three populations: non-mRFP (mRFPneg), low mRFP (mRFPlo), and high mRFP (mRFPhi) expressing cells (Figure 1D) were evident in three out of four cell lines. The number of mRFPhi cells was dependent on the cell line but ranged from 0.1 ± 0.0% for MDCC-WV5113 to 8.6 ± 2.1% for MDCC-KJ1063 cells in replicate experiments (Table 2). The percent of mRFP+ cells was considerably higher than previous reports on eGFP+ cells using vUL47eGFP (33, 34) and suggested this system may be used to better evaluate early stages of reactivation. Since MDCC-WV5113 failed to show a shift in mRFP, we excluded this cell line in future studies. These results show that mRFP expression can be used to quantify the reactivation of MDV in MDCCs *ex vivo*.

**Table 2:** Summary of mRFPneg, mRFPlo, and mRFPhi in Marek's disease virus (MDV)-transformed chicken cells (MDCCs).

**MDCC^a^**	**Viability (%)^b^**	**mRFPlo (% of cells ±SD)^c^**	**mRFPhi (% of cells ±SD)^d^**
KJ1072	95.6 ± 1.0	40.0 ± 17.5	2.5 ± 0.2
KJ1063	97.6 ± 0.8	55.1 ± 15.3	8.6 ± 2.1
WV5113	94.0 ± 1.9	1.6 ± 0.2	0.1 ± 0.0
WV6847	88.7 ± 2.0	31.3 ± 17.0	4.5 ± 2.1

^a^MDCC designation.

^b^Determined based on percent of cells negative for DAPI staining in flow cytometry.

^c^Percent (%) cells expressing low mRFP (mRFPlo) ± standard deviation (SD) in three biological replicates.

^d^Percent (%) cells expressing high mRFP (mRFPhi) ± standard deviation (SD) in three biological replicates.

### 3.3. Temperature treatment is superior to chemical treatment for reactivation of MDV

#### 3.3.1. Cell viability

Next, we examined mRFP expression during induced reactivation. Standard treatments to induce reactivation of herpesvirus-transformed cell lines include tetradecanoyl phorbol acetate or sodium butyrate treatment (48, 49). These treatments have been used to reactivate MDV in MDCCs (18–20); however, in our experience, the chemical treatment also causes significant cell death that affects downstream studies. Therefore, we sought to find alternative methods to induce the reactivation of MDV in MDCCs.

**Figure 2:**
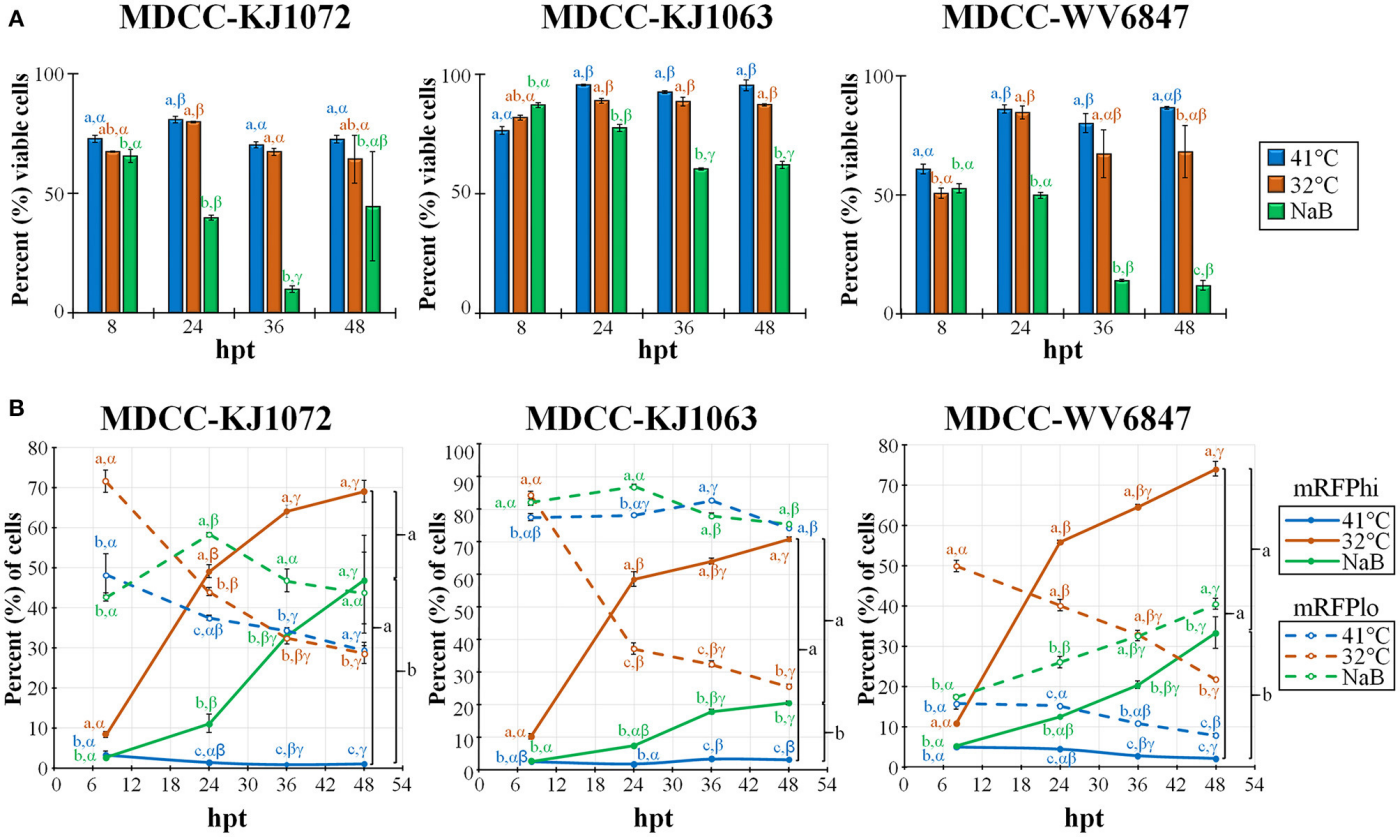
Cell viability and mRFP expression in three MDCC lines. **(A)** The percentage of viable cells was determined by using DAPI stain exclusion with flow cytometry in untreated (41°C), temperature (32°C), or sodium butyrate (NaB) treatment at 8, 24, 36, and 48 hpt. **(B)** Flow cytometry data of mRFPhi (solid lines) and mRFPlo (dotted lines) populations in all three cell lines after reactivation with temperature (32°C) and chemical (NaB) treatment. For both **(A, B)**, all data presented as mean ± standard deviation (*n* = 3/group) and analyzed by Friedman's test for two-factor analysis, followed by Nemenyi *post hoc* using R (Studio). Superscripts a, b, and c designate statistical differences (*P* < 0.05) between the treatments at the same time points, whereas Greek letters α, β and γ indicate statistical differences (*P* < 0.05) within time for the same treatment group. Colors are as in legend for each group.

Previous work showed that decreasing the temperature from 41°C to 37°C can induce reactivation of MDV (21, 22). Therefore, we tested temperature-induced reactivation using numerous treatment parameters and optimized an efficient and robust methodology to induce reactivation of MDV by decreasing the temperature to 32°C. A summary of this work is shown in Figure 2. Cell viability was measured using DAPI stain exclusion to differentiate live from dead cells (Figure 2A). The cell viability of the control (41°C) group for each cell line was consistently lower at 8 h post-treatment (hpt), likely due to hypoxia, serum starvation, and low temperature during Histopaque purification (16). When comparing each treatment for KJ1063, sodium butyrate (NaB) significantly reduced cell viability from 86.8 ± 1.0% at 8 hpt down to 77.3 ± 1.4, 60.3 ± 0.4, and 61.9 ± 1.5% at 24, 36, and 48 hpt, respectively. In contrast, cell viability of the low temperature (32°C) treatment group remained the same following 8 hpt (88.6 ± 1.0%, 88.3 ± 1.8%, and 87.0 ± 0.5% at 24, 36, and 48 hpt, respectively). Similar results were seen for all three cell lines.

#### 3.3.2. MDV reactivation

Next, we examined the percent of cells reactivating MDV based on the expression of mRFP using flow cytometry (Figure 2B). Again, all three cell lines performed similarly, although there were some differences in the total percent of cells with time and treatment groups. Simultaneously, the mRFPhi populations remained at approximately 2 to 5% in the untreated groups, while over time, the percent of cells expressing high levels of mRFP (mRFPhi) were significantly increased when treated at 32°C or with NaB compared to untreated cells (41°C). In all three cell lines, the percent of mRFPhi cells at 48 hpt was higher in 32°C treated groups than in NaB treatment. The increase of mRFPhi cells in the low-temperature treatment group was the highest among the three groups. As excepted with the rise in mRFPhi cells during 32°C and NaB, treatments, the percentage of mRFPlo cells decreased in all cell lines over time.

#### 3.3.3. Necrosis and apoptosis

**Figure 3:**
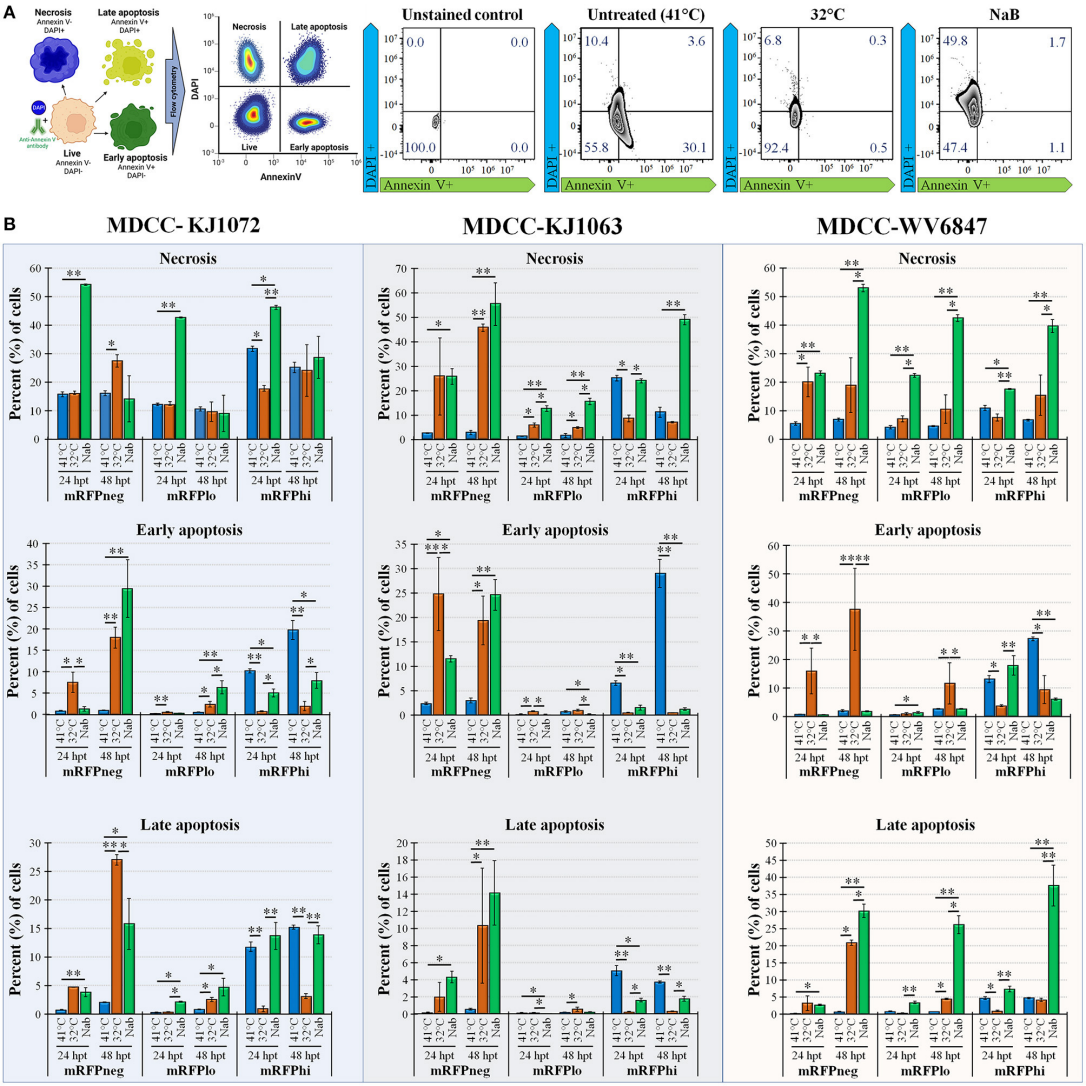
Necrosis and apoptosis after reactivation using Annexin V and DAPI staining. **(A)** Summary methodology to distinguish live, necrotic, and early vs. late apoptosis using Annexin V and DAPI staining. The cells can be differentiated at the stages of necrosis (DAPI+, Annexin V-), early apoptosis (DAPI-, Annexin V+), and late apoptosis (DAPI+, Annexin V+). **(B)** Summary of flow cytometry data for MDCC-KJ1072, -KJ1063, and -WV6847 with the percentage of necrotic, early apoptotic cells, and late apoptotic cells after reactivation. Data presented as mean ± standard deviation (*n* = 3/group). The three cell populations (mRFPneg, mRFPlo, and mRFPhi) are shown separately. Data presented as mean ± standard deviation. Significant differences using Kruskal–Wallis H test followed by Dunn's *post hoc* test with Bonferroni adjustment [treatment group (G) × time (T)] are shown (*p < 0.05; **p < 0.01; and ***p < 0.005).

Next, we examined apoptosis between temperature and NaB treatments during MDV reactivation. Using a combined Annexin V and DAPI staining method (50), cells were differentiated at the stages of necrosis (DAPI positive, Annexin V negative), early apoptosis (DAPI negative, Annexin V positive), and late apoptosis (DAPI positive, Annexin V positive). Live cells are DAPI and Annexin V negative. An example of this analysis is shown in Figure 3A using mRFPhi cells of cell line KJ1063 at 24 hpt. Using this approach, the stage of cells was determined for MDCC-KJ1072, -KJ1063, and -WV6847 cell lines for the three mRFP populations: mRFPneg, mRFPlo, and mRFPhi. The cells were analyzed at 24 and 48 hpt (Figure 3B) and separated based on necrosis, early apoptosis, and late apoptosis. All three cell lines exhibited similar trends; therefore, we used the KJ1063 cell line to illustrate the findings below.

Among the mRFPneg cells, there was a low percentage of necrotic, early apoptosis, and late apoptotic cells in the untreated (41°C) group. On the contrary, there was a marked increase of necrosis and apoptosis in the reactivation groups after 24 hpt. Interestingly, there was higher early apoptosis in the 32°C temperature treatment group at 24 hpt compared to the NaB treatment group, but the NaB treatment group had the highest percentage of necrosis and total apoptosis among all groups.

A similar result was seen in the mRFPlo population, with this population having the highest percentage of live cells among all populations and a significant increase in the percentage of necrotic cells after reactivation. The average percent (%) of cells in necrosis was 1.6 ± 0.8% in the untreated group, 4.8 ± 0.4% in the 32°C temperature group, and 15.5 ± 1.3% in the chemical treatment group.

In the mRFPhi population, 32°C temperature treatment had the lowest percentage of necrosis, early apoptosis, and late apoptosis, while the untreated control (41°C) group had the highest rate of early and late apoptotic cells. The chemical treatment group had marked increases in necrosis compared to untreated (41°C) and 32°C temperature treated groups at 48 hpt. Overall, the results show that temperature treatment resulted in a significantly lower percentage of necrotic and apoptotic cells compared to chemical treatment for all three cell lines.

### 3.4. Temperature treatment does not increase plaque counts in reactivation assays

Since temperature treatment appeared to result in reactivation of MDV in MDCCs, we wanted to quantify plaques produced following 32°C treatment. We hypothesized 32°C treatment would “prime” virus to reactivate more efficiently in reactivation assays. Table 3 summarizes these results using three MDCCs comparing plaques formed in CECs following 24 h treatment a 41°C or 32°C treatment. There were no significant differences for all three cells lines using Student's *t-*tests (*p* < 0.05) showing temperature treatment had no effect on “priming” MDV for reactivation.

**Table 3:** Temperature treatment on reactivation.

**MDCC^a^**	**Temperature^b^**	**PFU/10^5^ cells^c^**
KJ1072	41°	22.3 ± 3.8
	32°C	36.0 ± 19.6
KJ1063	41°C	29.5 ± 12.0
	32°C	30.5 ± 9.38
WV6847	41°C	263.3 ± 90.7
	32°C	220.0 ± 10.0

^a^MDCC designation.

^b^Temperature cells were incubated at for 24 h prior to reactivation assay.

^c^Plaque-forming units (PFU) per 10^5^ cells seeded.

### 3.5. MDV mRNA and protein expression during MDV reactivation

Herpesviruses are well-known for the temporal order of expression of genes in a cascade fashion. The overall cascade fashion of herpesviral gene expression has been previously described by Pellet and Roizman (47). Briefly, upon entry into the host cell, VP16 induces the expression of immediate early (IE) or alpha (α) genes Infected Cell Proteins (ICPs) ICP0, ICP4, ICP22, ICP27, and ICP47 (51, 52), which in turn activate early (E) or β genes through transcriptional activation. Early genes are involved in the initiation of the DNA replication and mRNA transcription complexes. Following amplification of the viral DNA genome, late (L) or γ gene products are produced and include all the gene products needed to produce a viral particle, including capsid and envelope proteins essential for infectivity. However, it is not completely understood how viral gene expression is initiated during reactivation.

**Figure 4:**
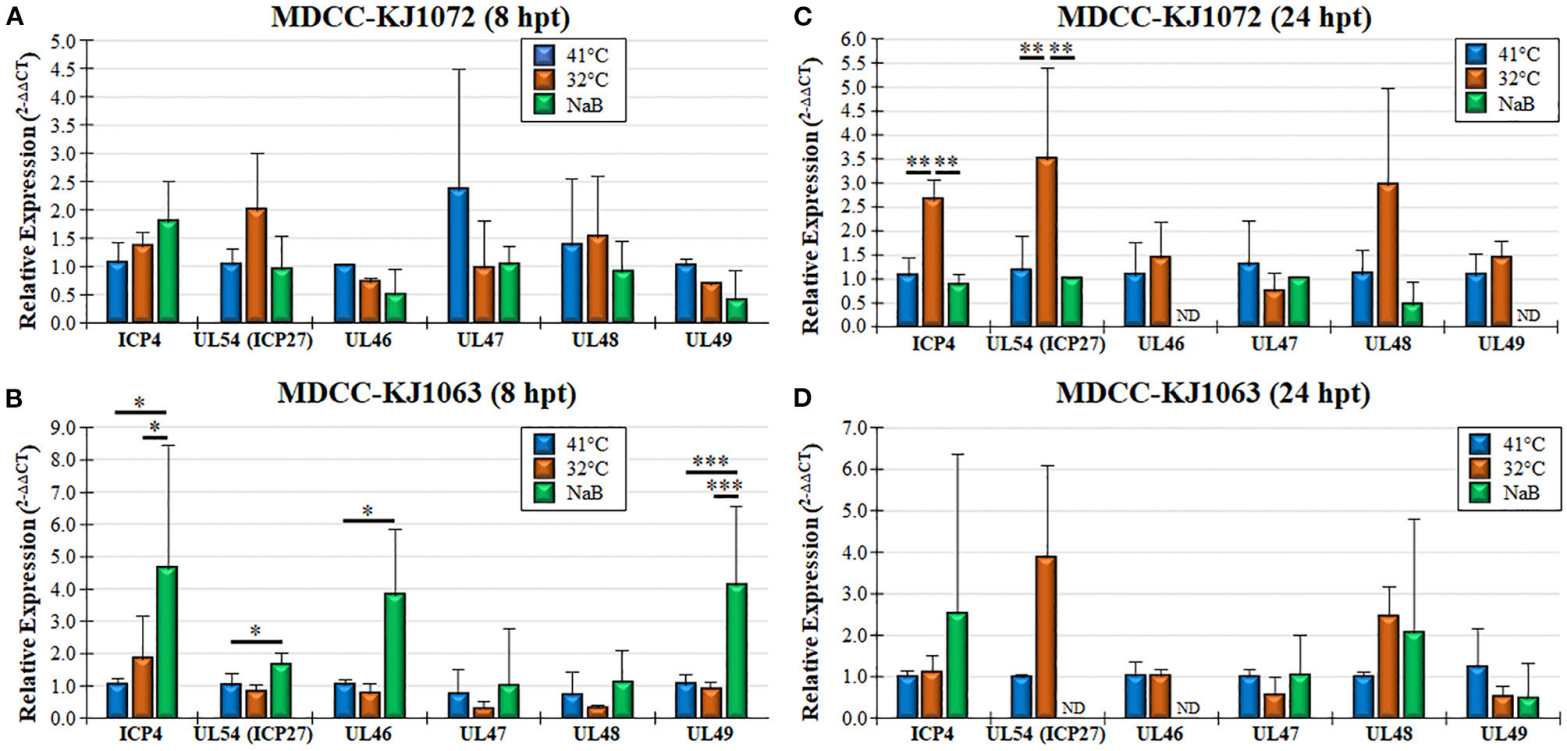
Viral gene transcription during reactivation using RT-qPCR assays. Total RNA collected from MDCC-KJ1072 **(A, C)** and –KJ1063 **(B, D)** at 8 **(A, B)** and 24 **(C, D)** hpt. The critical threshold (C_T_) values were used to calculate the mean viral mRNA fold changes in cells normalized to chicken GAPDH. Viral transcripts for ICP4, UL54 (ICP27), UL46, UL47, UL48, and UL49 were measured in triplicate using the ^2−ΔΔCt^ method with standard deviations. Significant differences are shown as *p < 0.05; **p < 0.01; and ***p < 0.005 using one-way ANOVA test followed by Tukey's *post hoc* test. ND = No endogenous gene transcription detected.

Treatment of MDCCs by decreased temperature or NaB treatment resulted in increased mRFP expression in cells that were presumed to be an indicator of MDV reactivation of viral gene expression based on staining for anti-MDV antigens, pp38 and gB (Figure 1B). To further examine viral gene expression during reactivation, we used RT-qPCR and western blotting for MDV genes. Since all three cell lines had similar responses, we only used MDCC-KJ1072 and -KJ1063 to analyze viral gene transcription at 8 and 24 hpt. Expression of immediate early (*ICP4, UL54*) and late (*UL46, UL47, UL48*, and *UL49*) viral genes were examined using RT-qPCR assays. Interestingly, the fold change in mRNA transcripts was modest and dependent on the cell line, with no significant increase for all genes for KJ1072 at 8 hpt (Figure 4A), while KJ1063 had significant increases in *ICP4, UL54, UL46*, and *UL49* transcription at 8 hpt following sodium butyrate treatment compared to the control group (Figure 4B). At 24 hpt, *ICP4* and *UL54* were significantly increased (*p* < 0.01) in MDCC-KJ1072 with 32°C treatment compared to 41°C and sodium butyrate treatment (Figure 4C). The chemical treatment group had high variability between 8 hpt and 24 hpt (Figures 4C, D), which was likely due to significant cell death at 24 hpt and poor RNA quality resulting in no detection of *GAPDH* transcripts (ND).

**Figure 5:**
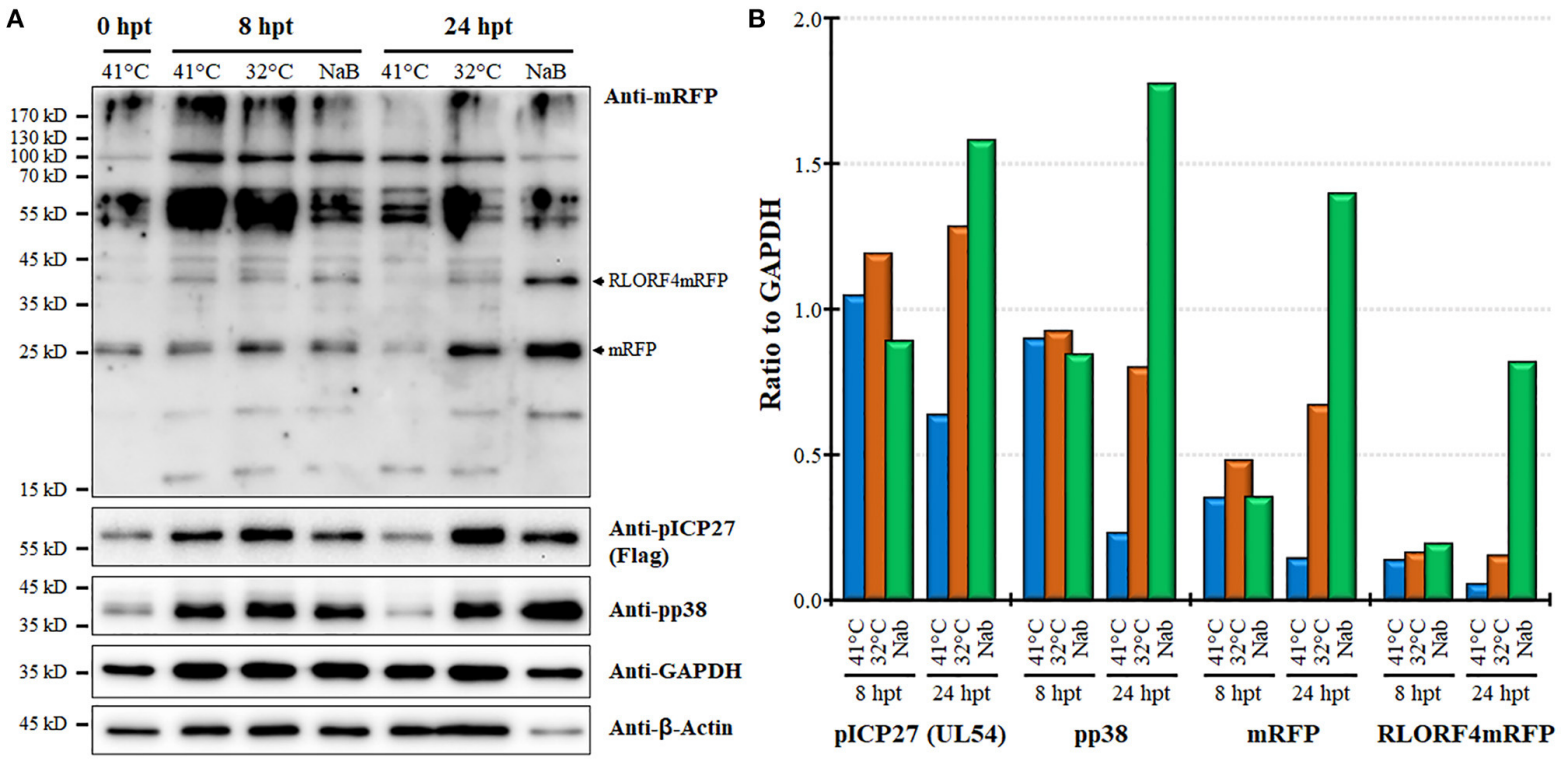
Viral protein expression during reactivation. **(A)** Western blotting was used to examine mRFP, pICP27 (UL54), pp38, GAPDH, and β-Actin at 8 and 24 hpt. Protein was also collected at time 0 hpt before treatment as a baseline control. RLORF4mRFP (~42 kD) and mRFP (~25 kD) bands are noted. **(B)** Densitometry was used to compare the ratio of viral proteins relative to GAPDH for each group.

Next, we examined protein expression at 8 and 24 hpt utilizing the MDCC-KJ1063 since it expresses a 3 × Flag tag on pICP27 for analysis (38). Western blotting for mRFP, pICP27 (UL54), pp38, β-Actin, and GAPDH are shown in Figure 5A. Multiple bands were detected using the anti-mRFP antibody. Previous reports have demonstrated significant alternative mRNA splicing within the repeat long regions (26, 53, 54), and these results suggest that at least some of the bands may represent splicing with the mRFP ORF at the C-terminus of RLORF4. Bands representing mRFP (~25 kD) and RLORF4mRFP (~42 kD) appear to increase during reactivation treatment, especially at 24 hpt. Similar results were seen for both pICP27 (UL54) and pp38 with both proteins increasing over time. Quantitative analysis of protein intensity showed little to no increase in pICP27, pp38, mRFP, and RLORF4mRFP at 8 hpt, while all were increased at 24 hpt (Figure 5B). NaB appeared to increase protein expression more than temperature treatment; however, this may be due to severe reduction in cellular GAPDH expression thus increasing the viral protein levels' ratio to GAPDH, that is evident in Figure 5A. In all, these results suggest that mRFP expression in live cells is a good indicator for MDV reactivation in MDCCs. Our results show that treatment of MDCC at 32°C results in abundant virus reactivation with less toxic effects than chemical treatment.

### 3.6. Time in culture (TIC)- dependent reactivation

During our initial studies on mRFP expression and reactivation of the four MDCCs, we noticed that the number of plaques generated in reactivation assays appeared to decrease over time. Former studies have shown similar observations (22) but those studies were more observational and not controlled comparing variability between cell lines and a range of time in culture. To confirm this observation in culture by comparing exact times in culture, plaques were quantified at 15 and 51 weeks for MDCC-KJ1072 and -KJ1063. Both cell lines showed significant decreases in the number of reactivating cells measured by plaque assays (Table 4).

**Table 4:** Spontaneous reactivation and time in culture.

**MDCC^a^**	**Time in culture^b^**	**Viability (%)^c^**	**PFU/10^5^ cells^d^**
KJ1072	15 wk	98.2 ± 0.2	48.4 ± 18.0
	51 wk	80.7 ± 2.9	25.5 ± 4.1*
KJ1063	15 wk	95.2 ± 0.8	99.5 ± 14.0
	51 wk	94.7 ± 0.7	32.8 ± 7.9*

^a^MDCC designation.

^b^Weeks (wk) in culture during characterization.

^c^Determined based on percent of cells negative for DAPI staining in flow cytometry.

^d^Plaque-forming units (PFU) per 10^5^ cells seeded.

^*^p < 0.05, Students t-test.

**Figure 6:**
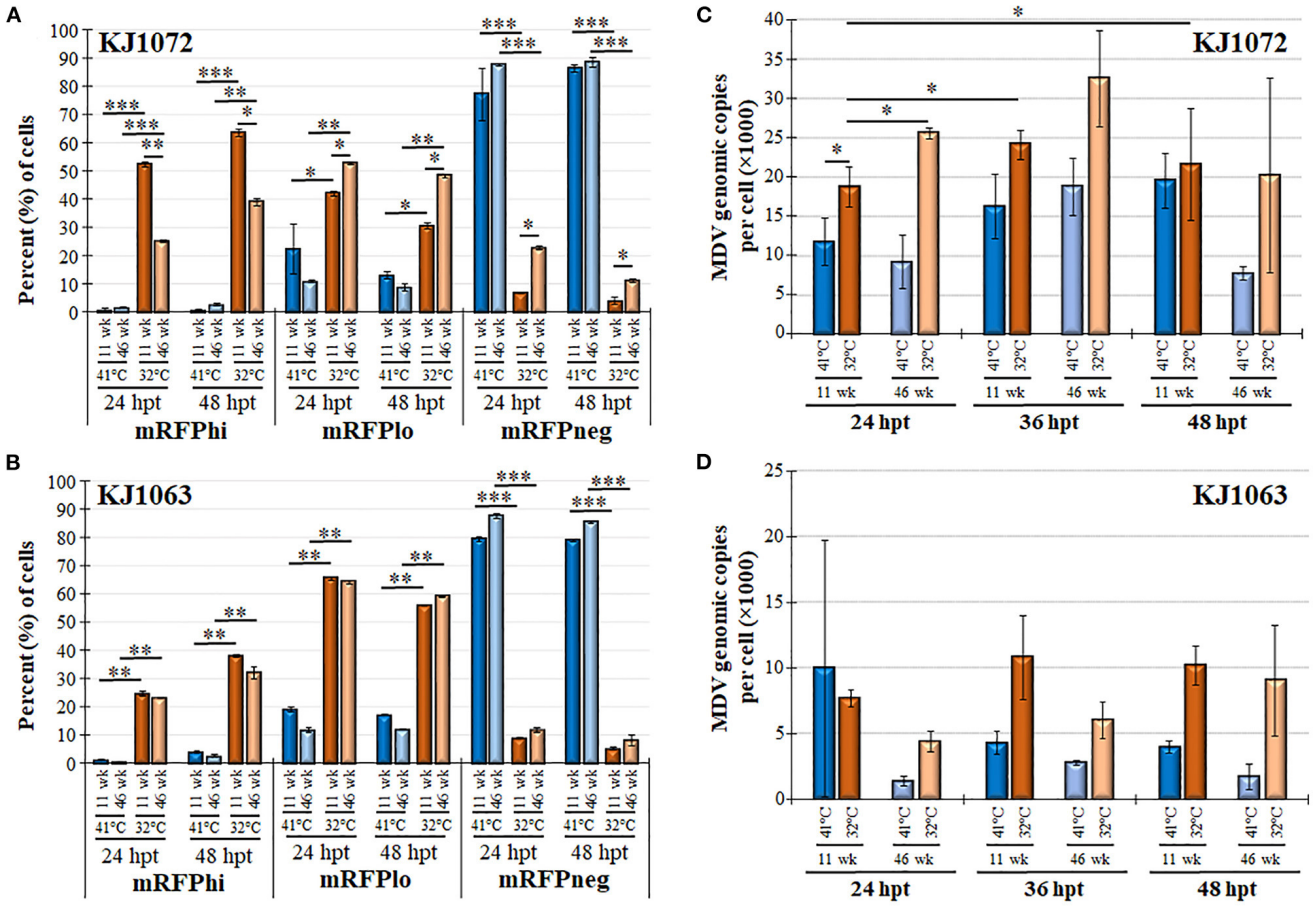
Decreased MDV reactivation in MDCCs with time in culture. The percent of cells expressing mRFP (mRFPneg, mRFPlo, and mRFPhi) at 11 and 46 wk in culture for MDCC-KJ1072 **(A)** and -KJ1063 **(B)**. Data presented as mean ± standard deviation. Significant differences are shown as *p < 0.05; **p < 0.01; ***p < 0.005 using Friedman's Test for two-factor analysis followed by Nemenyi *post hoc* using R (Studio) MDV genome copies were measured at 11 and 46 wk in culture following reactivation by 32°C treatment in MDCC-KJ1072 **(C)** and -KJ1063 **(D)** at 24, 36, and 48 hpt. Data presented as mean ± standard deviation (*n* = 3/group). Significant differences are shown as *p < 0.05; **p < 0.01; and ***p < 0.005 using 2-way ANOVA [treatment group (G) × time (T)].

Next, we examined mRFP expression during reactivation by temperature change comparing 11 and 46 weeks in culture for MDCC-KJ1072 (Figure 6A) and -KJ1063 (Figure 6B). The mRFPhi population was significantly higher at 11 wk in culture compared to 46 wk following reactivation at 32°C for both MDCC lines after 48 hpt. In contrast, there were significant decreases in mRFPlo and mRFPneg populations in the KJ1072 line, and a similar trend was found for KJ1063 but not statistically significant. Consistent with our current results, treatment of both cell lines at 32°C increased the mRFPhi and mRFPlo populations while lowering the mRFPneg percent of cells. Using qPCR, the MDV genomic copies per cell were measured at 11 and 46 wk in culture during reactivation (Figures 6C, D). Reactivation with 32°C treatment induced significant increases in viral genomic copies in MDCC-KJ1072 at 11 and 46 wk in the culture at 24 hpt (Figure 6C). However, there was no significant differences at 36 and 48 hpt. Interestingly, we saw no significant change in viral genomic copies for MDCC-KJ1063 (Figure 6D). In all, the results suggest that time in culture significantly affects the ability of MDV to reactivation *ex vivo*.

## 4. Discussion

Latency is a crucial feature shared among all herpesviruses. It helps keep the virus reservoir and to evade the host's immune system without destroying the host cell. In this report, we established two valuable tools for examining the reactivation of MDV in transformed MDCCs. First, we show that expressing mRFP within the repeat region of MDV allows an efficient method to evaluate live cells reactivating viruses. Second, we offer a less punitive technique to induce reactivation of MDV in MDCCs that results in significantly less necrosis and apoptosis, especially after 24 hpt. These novel tools will be valuable in studying the complex mechanism involved in herpesviruses' transformation, latency, and reactivation.

In our study, all four MDCC lines studied were CD4+CD8-, consistent with previous findings (55). It is important to note the variability between MDCCs generated. In our study, we used two different viruses that differed only in a 3 × Flag epitope at the N-terminus of the UL54 gene (pICP27). In the three cell lines generated from kidney tumors of chickens infected with vRLORF4mRFP/3 × Flag54 (38), one cell line (MDCC-WV5113) had very little mRFP expression and reactivation following 30 wk in culture, while two other cells lines (KJ1063 and WV6847) reactivated differently. Clearly, multiple factors will play a role in the ability to even establish MDCC lines *ex vivo*, including the strain of the virus and host genetics. Still, even when these are the same, differences can be seen. Calnek et al. (22) found similar results examining viral antigen expression in MDCCs generated from different MDV strains and chicken lines. The most widely used MDCC line was first developed in 1974, named MSB-1 (56). It was derived from the spleen of a chicken infected with the BC-1 strain of MDV and has been used by numerous researchers for reactivation studies. In addition, the region within the host where MDV integrates may also play an important part in the ability of MDV to reactivate. Kaufer et al. (12) found that removal of the MDV-encoded telomeric repeats severely affected the ability of MDV to integrate and transform cells and ultimately, this severely affected the ability of MDV to reactivate from latency. The exact locations of integration for the four cell lines examined in the current study have not been identified, but it is possible the site of integration may play a key role in their differences in reactivation. Overall, our results highlight the importance of examining multiple cell lines and noting the time in culture in *ex vivo* studies to ensure results are not unique to a specific cell line, such as is routinely done with the MSB-1 cell line.

Sodium butyrate treatment of cells results in hyperacetylation of histones, among many other cellular changes (57, 58). Former studies on MDV reactivation in MDCC lines showed sodium butyrate could upregulate viral gene and protein expression (18–20). The mechanism for induction of reactivation has yet to be fully understood. Still, increased cellular stress through histone modification is suspected to result in induced apoptosis (59) and G2/M cell cycle arrest (60), likely resulting in virus escape (reactivation) from the cell. However, chemical treatment is only one of the multiple mechanisms to induce reactivation or fully productive virus replication, including UV light, serum starvation, temperature changes, and hypoxia (16, 23, 24, 61, 62). The core body temperature of chickens is approximately 41°C, while the surface body temperature can fluctuate in ambient temperatures and range from 20 to 40°C (36). Previous studies in which incubation at 37°C with or without serum starvation also found reactivation based on viral antigen expression or seeding on CECs (12, 22, 23, 63); however, it is difficult to compare each study to ours with differing techniques, cell lines, and temperatures between these studies. We did not see significant increases in mRFP expression using serum starvation and 37°C treatment (Jarosinski and Tien, unpublished observation) but there were clear increases in mRFP expression (Figures 2, 4–6) following incubation at 32°C, as well as treatment of sodium butyrate. The increase in mRFP expression was consistent with the reactivation of the virus for both treatments, as evidenced by increased viral gene expression (Figures 4, 5) and genomic copies (Figure 6). However, 32°C treatment was significantly less punitive to cell viability (Figure 2), likely through less induction of necrosis and apoptosis, especially at 48 hpt (Figure 3). Overall, these data indicate the low-temperature treatment can induce MDV reactivation as effectively as sodium butyrate treatment, without harsh toxic cellular effects.

One unexpected result found in this study was the low level of viral gene transcription induced by both temperature and chemical treatment (Figure 4). The likely reason for this is the mixed population of cells in this experiment in which mRFPneg, mRFPlo, and mRFPhi cells were mixed in examining viral gene transcription using RT-qPCR assays. There is likely a low level of viral gene transcription from cells spontaneously reactivating in untreated (41°C) cells that may mask gene induction during reactivation, as well as altering of cellular gene transcription. We found sodium butyrate treatment affected the expression of GAPDH severely at 48 hpt, resulting in variability in this group. The effect of sodium butyrate treatment on GAPDH can also be seen in our protein expression analysis (Figure 5), in which GAPDH was reduced. We consistently found GAPDH and β-Actin protein levels to be severely reduced in the sodium butyrate group, amplifying the effect this treatment has on general cell health. Thus, the high viral protein expression in sodium butyrate-treated cells at 24 hpt is amplified by the decreased level of GAPDH levels (Figure 5B). It would be of interest to examine viral and cellular gene expression in the three mRFP populations separately in future studies to further characterize these distinct populations.

Another unexpected result we found in this study was the relatively low level of viral DNA replication following reactivation (Figure 6) that was partially dependent on the cell line. There were no significant increases in MDV genomic copies at all time points during 32°C treatment for MDV-KJ1063 (Figure 6D), while MDCC-KJ1072 had increased, but were relatively modest at ~2-3-fold increases over untreated (41°C) cells. Although not directly tested in our study, MDV VP22 (UL49) expression in cells can lead to severe DNA damage and S-phase cell cycle arrest (64). In future studies, it would be of interest to examine VP22 expression during 32°C treatment for reactivation of cells.

## 5. Conclusion

Collectively, we have demonstrated an easy, reproducible, and robust protocol for monitoring the reactivation of MDV in live MDCCs *ex vivo* using mRFP expression within the repeat long region and a less toxic treatment to induce MDV reactivation by decreasing temperature. This treatment induces reactivation similar to chemical treatment with sodium butyrate without significant cell death associated with sodium butyrate treatment. Furthermore, the time in culture for MDCCs significantly affects the ability of MDV to reactivate, which can also complicate studies in which MDCCs have been cultivated *ex vivo* for extended periods. These tools will be invaluable when addressing the role viral and cellular genes play during the “switch” from latency to reactivation of herpesviruses.

## Data availability statement

The original contributions presented in the study are included in the article/supplementary material, further inquiries can be directed to the corresponding author.

## Author contributions

KJ and Y-TT designed the study. Y-TT performed, collected data from the experiment, analyzed data, and wrote the original draft of the manuscript. HA performed statistical analyses. Y-TT, HA, and KJ revised the manuscript. All authors have read and approved the final manuscript.
